# Mie Voids for Single-Molecule Fluorescence Enhancement in Wavelength-Scale Detection Volumes

**DOI:** 10.3390/s25227033

**Published:** 2025-11-18

**Authors:** Ivan Kuznetsov, Fedor Shuklin, Evgeny Ryabkov, Elena Barulina, Andrey Petukhov, Denis G. Baranov, Alexander Chernov, Aleksandr Barulin

**Affiliations:** 1 Moscow Center for Advanced Studies, Kulakova Str. 20, Moscow 123592, Russia; 2 Russian Quantum Center, Moscow 121205, Russia

**Keywords:** fluorescence correlation spectroscopy, fluorescence enhancement, light confinement, large mode volume, confocal microscopy

## Abstract

Mie voids have been recently demonstrated as a promising nanophotonic platform for light manipulation and optical sensing. Moreover, the detection volumes of Mie void cavities exceed those of optical nanoantennas, making them appropriate for low-concentration single-molecule fluorescence biosensing. However, the fluorescence enhancement quantification of diffusing molecules in such optical antenna systems has not been addressed. Here, we explore Mie void ability to enhance the single-molecule fluorescence of diffusing fluorophores AF647 with the help of fluorescence correlation spectroscopy. The optimized structure confines 635 nm laser light within a well-defined excitation volume in the Mie void and provides the excitation intensity enhancement. We monitor the reduction in the number of molecules, signifying the detection volume reduction in the Mie void and an increase in single-molecule brightness up to 2.8 times. However, we reveal that the observed fluorescence enhancement appears limited owing to the azimuthally symmetric emission direction away from the optical axis when the molecules diffuse in the vicinity of the Mie void entrance. Altogether, this study demonstrates exploration of Mie void-based nanoantenna potential for single-molecule fluorescence spectroscopy applications.

## 1. Introduction

Single-molecule fluorescence spectroscopy provides unprecedented capabilities for the optical observation of molecules within a natural aqueous medium and under ambient conditions [1,2,3]. Specifically, the monitoring of fluorescence fluctuations from individual molecules represents an effective methodology for determining molecular concentrations [4] and interrogating fast interaction and conformational dynamics [5,6]. Fluorescence correlation spectroscopy (FCS) facilitates the monitoring of numerous single-molecule events over the acquisition timeframe, enabling the capture and interpretation of the system’s most significant fluorescence fluctuations. FCS enables the determination of the number of molecules, their sizes, and monitors binding interactions, which could be potent as a pathology analysis method [7,8,9]. The efficacy of such single-molecule analyses would be significantly augmented by fluorescence enhancement, as it would permit faster event capture and reduce overall analysis time [10,11].

Confinement of optical radiation is one of the key requirements for fluorescence enhancement. Various platforms for light confinement include but are not limited to diffraction-limited optical microcavities [12,13,14] as well as surface plasmon-polariton structures [15,16,17]. The use of optical plasmonic nanoantennas are a well-established technology for achieving substantial fluorescence enhancement through the confinement of light within a localized “hot spot” [18,19,20,21,22,23,24]. A key advantage of these structures is their small optical mode volume, which is particularly advantageous for studying single molecules at high concentrations for molecular dynamics studies [25,26]. However, these nanoantenna configurations are subject to significant limitations, as the nanogap antennas typically exhibit considerable Ohmic losses due to the close proximity of the emitter to the metal surface [18,19,27,28]. Furthermore, the dimensions of the enhanced-field region or a “hot spot” are generally insufficient to accommodate large nanoparticles [29,30,31], as even zero-mode waveguide size typically resides at about 100 nm [19,28,32]. All-dielectric spherical meso-sized cavities theoretically provide high fluorescence enhancement factors inside and outside the spheres [33]. Alternatively, annular Bragg resonant cavities provide annular light focusing, resulting in fluorescence enhancement in a central “hot spot” [34,35]. These constraints hinder the application of plasmonic nanoantennas in diffusion-based biosensing at the single-particle and single-molecule levels, where a large detection volume and a low-loss resonator are often preferable for comprehensive analysis [36,37,38]. For example, achieving single-molecule sensitivity is critical for the accurate analysis of cargo molecules in extracellular vesicles like exosomes. Techniques such as fluorescence burst analysis require the precise determination of burst location with microsecond temporal accuracy [39,40] to validate the coincidence of events in multiple color channels. While large-mode-volume photonic structures, such as photonic crystal cavities, offer an alternative for fluorescence enhancement [41,42], their implementation is often hampered by complex design and fabrication requirements, in addition to a typically narrow operational bandwidth. Hybrid plasmonic–dielectric systems feature potential for light manipulation within the nanoantennas with reduced fluorescence quenching [43]; however, typically, the cavities are still associated with nanometer-scale gap sizes [44,45].

In contrast, voids inside high-index dielectric materials, known as Mie -voids, present a class of resonant nanophotonic structures capable of confining light externally to the dielectric host medium, due to refractive index contrast. Theoretically studied decades ago [46,47,48], Mie voids have recently re-emerged with experimental implementations in silicon [49] and van der Waals materials [41]. The presence of losses in the host medium results in drastic quality factor enhancement in the vicinity of resonance and, as a consequence, stronger light confinement within the voids [50]. Utilization of high-loss materials naturally pushes the operational spectral range to UV. Alongside diameter- and depth-dependent resonant behaviors, these unique properties make Mie voids a robust and multifaceted platform for manipulation of light–matter interactions, including applications in nanoscale color printing [40,42] and sensing [43]. With characteristic mode volumes on the order of a wavelength [43], they hold promise for emission-enhancing sensors with large detection volumes. However, their potential for boosting fluorescence, to the best of our knowledge, has yet to be experimentally realized.

In this work, we explore Mie voids as a novel class of optical antennas for single-molecule fluorescence sensing at an excitation wavelength of 635 nm. The confocal microscope’s focal spot was precisely aligned with the Mie void to efficiently couple the laser excitation. A moderate numerical aperture (NA = 0.6) objective lens was utilized to simultaneously provide a large detection volume suitable for biosensing and fully cover the Mie void cavity with the focal beam. The designed Mie voids effectively confine light within their cavity when immersed in an aqueous solution containing fluorescent molecules or nanoparticles. Using fluorescence correlation spectroscopy (FCS), we confirm a significant reduction in the effective detection volume and a concomitant increase in single-molecule fluorescence brightness. The observed fluorescence enhancement reaches a factor of approximately 2.8 compared to a standard glass slide and 1.4 relative to a silicon reflecting substrate. These results, which account for the excitation gain, quantum yield gain, and the large confocal volume relative to the Mie void size, are in agreement with numerical simulations. Furthermore, we successfully extend the application of the Mie void platform to large emitters, including streptavidin-conjugated AF647 molecules and quantum dots. Altogether, these findings demonstrate the significant potential of Mie voids for fluorescence-enhanced biosensing applications down to the single-molecule level.

## 2. Materials and Methods

### 2.1. Design and Fabrication of Mie Voids

The optimization of Mie void geometry via maximizing pump field confinement in a single spot was carried out via FDTD simulation software (Ansys, Canonsburg, PA, USA) (Figure S1). The Mie voids wererepresented by nanowells on the silicon surface with a defined radius and depth. By monitoring the axial electric field profile inside the Mie void, we revealed the region of suitable geometries with a well-confined excitation volume at a laser wavelength of 635 nm that dominates over other hot-spots near the silicon–water interface. Via confining light in a single spot in the cavity, the fluorescence correlation spectroscopy formalism of single-molecule 3D Brownian diffusion in Gaussian-like focal volumes can be applied similarly to plasmonic and dielectric nanoantennas [18,29], and zero-mode waveguides [35,51]. In such a structure, emission enhancement is mainly owing to (i) molecule absorption enhancement proportional to excitation field intensity, and (ii) Purcell emission enhancement stemming from the local density of optical states (LDOS) peak in the vicinity of a resonator. While the optimization of the geometry has been performed via the FDTD simulation of the pump intensity, the Purcell factor and quantum yield enhancement within the AF647 fluorescence detection wavelength band are obtained through quasi-normal mode (QNM) calculations. Therefore, the absorption enhancement and Purcell factor can be obtained directly by solving for QNMs of the void in COMSOL 6.1 with eigenfrequencies within excitation and absorption regions [52], estimating field confinement from QNM field patterns in addition to FDTD results and Purcell enhancement via LDOS ratio $F_{P}(r_{e},\;\omega )=\rho (r_{e},\omega )/\rho_{0}(r_{e},\omega )$, where $r_{e}$ is the position of the molecule within the void, and ρ and $\rho_{0}$ are respectively LDOS with and without voids [53]. LDOS, corresponding to an isotropic emitter, can be obtained through the optical theorem as the imaginary part of the trace of Green’s tensor. In turn, Green’s tensor can be obtained with eigenmode decomposition. This approach enables relatively easy calculation of absorption and emission enhancement as an eigenfrequency problem with PML boundaries, and averaging over the void volume is more reliable, in contrast to calculating Purcell enhancement via dipole emission power (see Supplementary Materials, Supplementary Note S1). Furthermore, this approach enables calculation of quality factors and mode volumes of the leading QNMs, resulting in the *Q* factor of 27.9 of the mode, closest to the emission peak, and generalized mode volume, averaged over the void volume defined as in [54], $V\;=\;1.81\cdot 10^{-17}+\;i\cdot 1.5\;\cdot \;10^{-17}$ m^3^. Note that the *Q* factor is rather low, but still comparable to leaky Mie cavities and Mie modes, while the mode volume is considerably larger than that in a typical leaky scatterer, due to the placement of the void on the interface of two media, one of which has no refractive index contrast with the void. The same order of real and imaginary parts of the mode volume points to string energy leakage. Such differences in *Q* and *V* affects the Purcell enhancement, as Purcell factor F~Re(Q/V). Comparing the mode volume parameter to that of other microresonator platforms, e.g., Fabry-Perot microcavities, the latter exhibit extremely high finesse, as well as mode volumes of thousands of femtoliters (1 fl = 10^−18^ m^3^) with extremely high *Q* factor [12,55]. On the other hand, the mode volume of whispering gallery modes is tens of fl for near-IR wavelengths [56], which turns out to be comparable with the deduced mode volume of the Mie void. Photonic crystal cavities can shrink mode volumes to the wavelength-scale of sub-femtoliter volume in the optical wavelength range [57]. Experimentally, fluorescence enhancement factors in wavelength-scale microresonators typically may reach up to 1 order of magnitude for low or modest quantum yields [58]. The improvement of quality factors becomes limited after reaching a strong coupling regime [41]. While nanogap nanoantennas may ensure large enhancement factors depending on the fluorophore quantum yield, the mode volume can normally shrink to the zeptoliter range in the visible [59,60] or even down to 0.04 zl for nanoparticle-on-mirror platforms [61].

Silicon wafers with a resistivity of 0.005 Ohm·cm were utilized as host media for Mie voids, which can be easily visualized through scanning electron microscopy (SEM). The wafers were diced into 1 cm square silicon substrates with sides of 1 cm. Mie voids were carved with a focused-ion beam microscope combined with SEM (FIB-SEM, HELIOS 660). The FIB-SEM setup emits gallium ions at a current value of 18 pA. The correct current selection and the pressure minimization down to 10^−5^ Pa are required to make rather straight side walls with a constant diameter. The current values up to 89 pA produce smooth Mie void side walls, making the output geometry less predictable, as well as textured side walls. Clusters of 3 by 3 Mie voids with a pitch size of 10 μm were prepared for the excitation/emission characteristics of our home-built confocal fluorescence microscope and the AF647 benchmark fluorophores (Lumiprobe, Moscow, Russia).

### 2.2. Single-Molecule Detection Setup

The excitation light source is a continuous wave laser of 635 nm (Lasermasters, Moscow, Russia). The laser light was filtered through a short-pass filter and then was reflected by a dichroic mirror (DM10-638LP, LBTEK, Shenzhen, China) toward a stationary microscope body. The incident laser power is measured after the reflection from the dichroic mirror. A mounted objective lens (NA = 0.6, air immersion, OPTO-EDU, Beijing, China) was used to focus light into the fluorescent solution and the Mie voids. A 3-axis piezo stage (Nano-T225, MadCityLabs, Madison, WI, USA) was leveraged to accurately focus the beam onto a Mie void. The aqueous solution was prepared in the PDMS wells on top of the Mie void substrate and covered by a glass coverslip to prevent leakage. The fluorescence light was collected by the same objective lens and passes through a dichroic mirror, where the excitation and emission lines are decoupled. A long-pass filter (GCC-211106, Daheng Optics, Beijing, China) and a band-pass filter 660–740 nm (GCC-203006, Daheng Optics, Beijing, China) were incorporated to filter the target fluorescence emission and minimize the background noise level. The fluorescence light was spatially filtered via focusing light onto a pinhole of 80 μm with the tube lens (achromatic doublet with focal length of 200 mm, GCL-010606, Daheng Optics). Then, the light was collimated and tightly focused onto the active area of a single-photon counter (SPCM-AQRH, Excelitas, Pittsburgh, PA, USA). Photon time-tagged data were generated from TTL electrical pulses delivered from the single-photon counters to the time-tagging electronics module (Time Tagger Ultra Value, Swabian Instruments, Stuttgart, Germany) with a time resolution of 42 ps. The module was synchronized with the piezo stage with a position-change marker signal to perform high-magnification imaging with nanometer-scale step sizes. The collected photon time-tagged data were analyzed and fitted in Python 3.12.3-based programs (Visual Studio Code 1.106, Microsoft, Redmond, WA, USA).

AF647 is an organic fluorophore with the structure of C_39_H_44_N_3_K_3_O_16_S_4_, purchased from Lumiprobe (Moscow, Russia). The solutions of AF647 were prepared from a concentrated stock by diluting it in phosphate buffer saline (PBS). The streptavidin-AF647 conjugates were purchased from ThermoFisher Scientific (Waltham, MA, USA) and diluted in PBS. The CdSe/ZnS quantum dots were purchased from ThermoFisher Scientific (Waltham, MA, USA) and diluted in deionized water.

## 3. Results

**Figure 1:**
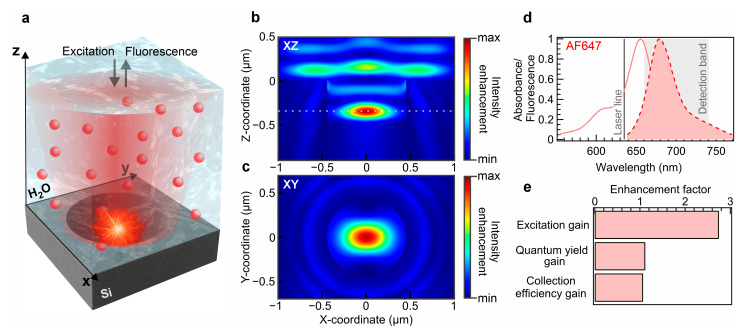
Mie voids for single-molecule fluorescence enhancement. (**a**) Schematic of Mie void accommodating single AF647 diffusing molecules within the mode volume. (**b**) Simulation results of the intensity enhancement within the Mie void in the XZ plane. The incident plane wave is polarized along the X-axis. (**c**) Simulation results of the intensity enhancement within the Mie void in the XY plane indicated by the dashed line in panel (**b**). The results of (**a**,**b**) are represented for a 635 nm wavelength. (**d**) Absorbance and fluorescence spectra of AF647. The gray line designates the employed laser wavelength, while the shaded region corresponds to the detection band of the fluorescence. (**e**) Computed enhancement factors, averaged across Mie void volume: excitation gain, quantum yield gain within fluorescence detection band, and collection efficiency gain.

The principle of the Mie void sensor application is shown in Figure 1a. As molecules diffuse into the Mie void cavity, they experience enhanced excitation within the modal volume. The cavity’s geometry is optimized to support a single resonant mode, which serves to precisely define the effective detection volume. Notably, the confined mode is localized near the center of the cavity (Figure 1b,c), in contrast to plasmonic nanoantennas, where the hot spot is typically constrained to the metal surface. In such metallic structures, a substantial portion of energy is dissipated non-radiatively into the metal, which inherently limits the maximum achievable enhancement, particularly for emitters with high intrinsic quantum yield. Furthermore, the mode volume of the Mie void is sufficiently large to accommodate nano-objects several hundred nanometers in size, such as quantum dots, polymeric nanoparticles, or exosomes [39]. The simulated field distribution exhibits a characteristic electric dipole emission pattern. A significant fluorescence enhancement is anticipated for emitters within the Mie void when compared to both a standard glass substrate and a silicon reflecting surface. The fluorescence enhancement ($\eta_{F}$) in the presence of the Mie void resonator is expected to be represented as follows [51]:

(1) $$\eta_{F}=\frac{\eta_{coll}\eta_{exc}\eta_{\Gamma_{rad}}}{\eta_{\Gamma_{tot}}}$$

where $\eta_{coll}$ denotes gain in collection efficiency, $\eta_{exc}$ is excitation gain, $\eta_{\Gamma_{rad}}$ representsradiative rate enhancement, and $\eta_{\Gamma_{tot}}$ denotes the total rate enhancement. Given that the total rate ($\Gamma_{tot}$) in the absence of a resonator can be represented as $\Gamma_{tot}=\Gamma_{rad}+\Gamma_{nr}$, where $\Gamma_{rad}$ and $\Gamma_{nr}$ are the radiative and non-radiative rate of the fluorescent emitter, respectively. The excitation gain is considered to be identified via the intensity enhancement in the confocal volume of a Mie void. Considering the light confinement averaged across the Mie void volume, we deduce an excitation gain of 2.75. The Purcell factor represents the metric of the influence on the radiative and non-radiative rate enhancement which enables deduction of a quantum yield enhancement of AF647 ($\eta_{Q}=\eta_{\Gamma_{rad}}/\eta_{\Gamma_{tot}}$) in the presence of a Mie void. Given the emission properties of the analyzed fluorophore (Figure 1d), the Purcell factor exhibits values up 1.6 above the excitation wavelength (Figure S2). Given the evolution of the quantum yield gain as a function of the Purcell factor, we determined a quantum yield enhancement of about 1.12 times. As for the collection efficiency, we determined the ratio of maximum power of the z-propagating waves emitted by a dipole in the center of the Mie void mode volume and the above the silicon substrate (Figure 1e). The directionality of the emission is optimal for the collection by the objective lens when the dipole is within the excitation volume (Figure S3). The collection efficiency gain was verified through sweeping the strictly upward emission for dipole in mesh nodes within the Mie void volume. The collection efficiency at the cavity entrance drops drastically, and recovers slowly upon the dipole lifting above the Mie void. Considering large detection volume constrained by the microscope point spread function size and the pinhole-based spatial filtering, the overall fluorescence enhancement that we could experimentally detect was approximated by ~3 times as compared to the glass slide reference.

**Figure 2:**
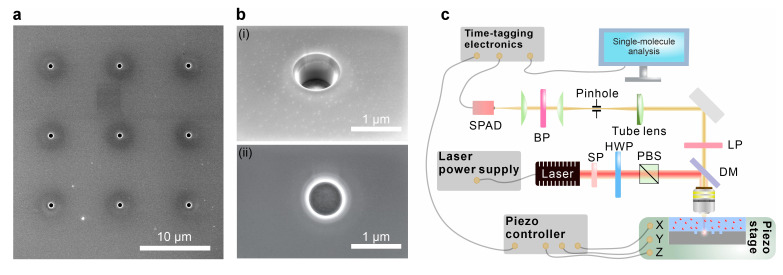
Mie void application for epi-fluorescence microscopy. (**a**) Scanning electron microscopy image of the Mie void cluster. (**b**) SEM of one of the Mie voids (**i**) at the oblique angle view (52°) and (**ii**) from the top view. (**c**) Fluorescence confocal microscope with time-tagging electronics to detect single-molecule analysis (in particular, fluorescence correlation spectroscopy). SPAD: single-photon avalanche diode; BP: band-pass filter; SP: short-pass filter; LP: long-pass filter; DM: dichroic mirror; PBS: polarizing beamsplitter; HWP: half-waveplate.

The optimal geometrical parameters for the Mie void consist of a depth of 480 nm and a radius of 450 nm. This size is sufficient to accommodate most exosomes found in biofluids—though not larger entities such as microvesicles or apoptotic bodies—thereby functioning as a large-mode-volume resonator capable of achieving a low molar limit of detection in optical single-molecule sensing. Focused ion beam (FIB) patterning was used to accurately control the size and depth of the Mie voids and to fabricate arrays suitable for statistical analysis (Figure 2a). The actual dimensions of the fabricated structures averaged 447 nm in radius and 488 nm in depth (Figure 2b). These were further characterized using optical microscopy. For single-molecule fluorescence measurements, a 500 pM solution of AF647 was deposited onto the Mie void substrate. A moderate numerical aperture (NA = 0.6) objective lens was employed to focus the excitation light and collect emissions from the confocal volume. A pinhole placed in the conjugate plane of the detection path provided spatial filtering (Figure 2c). Although a higher-NA objective would offer greater sensitivity, the resulting reduction in detection volume increases the limit of detection, making such configurations less suitable for sensing at pM or fM concentrations [62]. A 635 nm laser beam was focused through the sample onto the silicon interface. Using a piezo nanopositioning stage, individual Mie voids are located by identifying global changes in AF647 fluorescence signal in the Mie void center.

**Figure 3:**
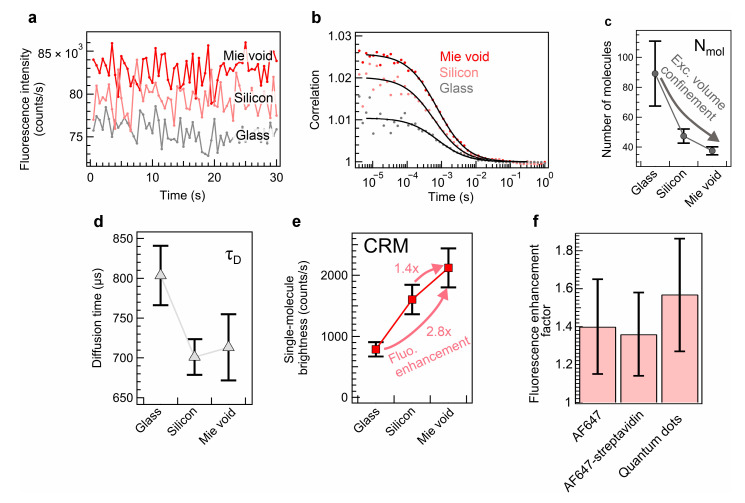
Mie void-enhanced FCS of diffusing AF647. (**a**) Fluorescence time traces of AF647 at 500 pM concentrations. (**b**) FCS autocorrelation functions retrieved from time-tagged data corresponding to panel (**a**). (**c**) Number of molecules change in the presence of glass slide, on the silicon substrate without Mie void and after localization of Mie void. (**d**) Diffusion time change in the presence of glass slide, on the silicon substrate without Mie void and in Mie void. (**e**) Diffusion time change in the presence of glass slide, at the bare silicon substrate and in the Mie void. The increase in single-molecule brightness corresponds to the fluorescence enhancement. Error bars for data points in panels (**c**–**e**) correspond to the standard deviations of the FCS measurements. (**f**) Experimental fluorescence enhancement factors in Mie voids from diffusing AF647, streptavidin conjugated with AF647, and quantum dots CdSe/ZnS.

The photon time-tagged data were recorded by the time-tagging electronics module connected to a single-photon avalanche diode. The fluorescence time traces of AF647 diffusing though the Mie void demonstrate higher average intensity as compared to the case of focusing onto the bare silicon interface (Figure 3a). Moreover, a silicon interface seems to enable higher fluorescence intensity as compared to a conventional glass slide case, because of the collection efficiency gain in fluorescence back-reflection by silicon (reflectance of ballistic photons of ~62% within the detection band). We computed the autocorrelation function from the photon time-tagged data to monitor the diffusion of AF647 molecules through the detection volume as follows:

(2) $$G\left(\tau \right)=\frac{\langle F\left(t\right)\cdot F\left(t+\tau \right)\rangle }{{\langle F\left(t\right)\rangle }^{2}}$$

where τ is the lag time values, *F*(*t*) is fluorescence intensity as a function of time, and brackets 〈 〉 denote the average operator. In the case of a Mie void, we monitored the increases in the autocorrelation amplitudes at near-zero lag times for the same molecule concentrations, which corresponds to the shrinkage of the mode volume within the Mie void cavity (Figure 3b). Also, the signal-to-noise ratio (SNR) of the autocorrelations increases as compared to the glass and silicon slide cases. The autocorrelation SNR is proportional to single-molecule brightness, as follows [63]:

(3) SNR=CRM2T∆τ(Nmol⋅CRM+B)1+1Nmol

*CRM* stands for count rate per molecule (single-molecule brightness), *T* is acquisition time, ∆*τ* is time channel width, $N_{mol}$ is the number of fluorescent molecules present in the detection volume, and *B* is background noise intensity. The background noise intensity does not exceed 5% of the total fluorescence signal (N⋅CRM+B); therefore, we omitted the influence of the background in the further experimental data analysis. Therefore, the reduction in autocorrelation function noise serves as a confirmation of the single-molecule brightness enhancement in the presence of the Mie void cavity. We fitted the autocorrelation functions through the three-dimensional Brownian diffusion model, as follows:

(4) $$G\left(\tau \right)=\frac{1}{N_{mol}}{\left(1+\frac{\tau }{\tau_{D}}\right)}^{-1}{\left(1+\frac{\tau }{{\kappa^{2}\tau }_{D}}\right)}^{-0.5}$$

where $\tau_{D}$ is the diffusion time and, κ is the detection volume aspect ratio. The FCS-determined number of molecules represents an effective metric to assess either the molecule concentration or the detection volume. When the silicon substrate is used, the number of molecules reduces as the measurement effectively occurs exactly at the reflective interface with present light confinement at the surface (Figure 3c). Meanwhile, in the case of a Mie void, the light is further confined within the void cavity, leading to further reduction inAF647 molecule number in the detection volume. Although the apparent Mie void-induced detection volume reduction is about 30% as compared to bare silicon, the number of molecules within the inherent confocal volume still contributes significantly to the determined value. The diffusion time shows a decrease with the silicon case as compared to free solution on a glass slide, which we attribute to the significant reduction in the detection volume dimension along the optical axis (Figure 3d). A Mie void does not seemingly reduce the diffusion time further as compared to the silicon substrate. We attribute this to a slight deviation in stochastic 3D Brownian diffusion, as the molecules reaching the mode volume can linger at the well sides before they leave the cavity, which leads to a slight slowdown of the diffusion. Nevertheless, it allows tracking of the emitter presence for a longer time, which improves the accuracy of the analysis and does not undermine the limit of detection. As FCS is an inherently single-molecule analysis relying on the fluorescence fluctuation monitoring within the mean signal, we can directly find the single-molecule brightness enhancement. *CRM* is deduced via the ratio of $\langle F(t)\rangle /N_{mol}$ in the bulk of the fluorescence solution, at the silicon interface, and in the Mie void cavity. The single-molecule brightness is enhanced from 0.8 kcounts/s by 2 times in the presence of silicon. The Mie void adds an extra enhancement factor of 1.4 with respect to the silicon substrate (Figure 3e). One of the reasons for the moderate enhancement factor value seems to stem from the loss of the collection efficiency at the Mie void entrance and above it (Figure S3). The emission pattern appears toroidal in the vicinity of the Mie void entrance, which is non-optimal for efficient fluorescence collection. Whereas bare silicon exhibits ordinary emission when the dipole is placed above the interface. Experimentally, a majority of fluorophores within the confocal volume are present above the Mie-void; therefore, their emission is preferentially sorted away from the optical axis direction. Such emission patterns lead to the reduction in the emission intensity outside the Mie-void mode volume; the molecule emission detection efficiency might be dumped outside the mode volume, leading to bright edges and the center of the Mie void with the toroidal dip of the signal intensity in between (Figure S4). Moreover, we demonstrate that a similar enhancement factor can be maintained by measuring the single-molecule brightness of streptavidin-AF647 conjugate biomolecules (size of ~5 nm) and quantum dots of core–shell structure CdSe/ZnS of sizes ~10–20 nm (Figure 3f). Therefore, we demonstrate the experimental validation of fluorescence enhancement in non-quenched fluorophores in Mie voids with large mode volumes.

## 4. Conclusions and Discussion

We demonstrate a Mie void antenna application for fluorescence enhancement of diffusing non-quenched fluorophores AF647 in aqueous solution. The size of the Mie void is suitable to accommodate nanoparticles of a few hundred nanometers in size and induce an excitation gain within one intense wavelength-scale excitation volume at the laser wavelength. The Purcell factor enhancements within the band allow for accelerating the photodynamics of molecules and promoting the radiative rate. Additionally, the collection efficiency within the Mie void is enhanced in the vicinity of the Mie void, while the emitters above the Mie void undergo a drastic reduction in collection efficiency that diminishes the total fluorescence enhancement factor, making the single-molecule fluorescence brightness similar to a bare silicon reference. Nevertheless, the single-molecule brightness remains significantly higher with respect to the glass reference. The large mode volumes, signal enhancement, and facile operation at pM concentrations make the platform rather applicable for biosensing applications of low-concentration or large objects, e.g., exosomes or plastic nanoparticles. Although plasmonic nanogap antennas exhibit substantially larger enhancement factors [18,64], the gap sizes appear too small to accommodate large nanoobjects, which could be relevant for sensing applications of organic and inorganic species. Moreover, nanogap antennas provide extremely confined mode volume, making them applicable for molecular dynamics monitoring at micromolar concentrations [25,65], rather than for low-concentration sensing. We utilize a modest NA objective lens with about a 2 μm focal spot size. In principle, a higher NA objective lens could be employed to enhance excitation intensity and collection efficiency for single-molecule fluorescence; however, the confocal volume should still be larger than the Mie void to preserve the fluorescence enhancement effect and keep a low limit of detection for the system. Considering strategies to improve directionality toward a collecting lens or modify the dielectric material may lead to further fluorescence enhancement in Mie void resonators. Altogether, Mie void antenna formalism enables an alternative single-molecule enhancement platform for the characterization of large nanoobjects at ultimate sensitivity. The azimuthally symmetric emission at the Mie void entrance limits the fluorescence enhancement factor when measured from single diffusing molecules. Such a methodology may be applied for precise single-molecule counting within single particles, or for fast fluorescent nanoparticle detection and characterization in the framework of precision medicine and environmental monitoring.

## Data Availability

Data are contained within the article.

## Supplementary Materials

The following supporting information can be downloaded at: https://www.mdpi.com/article/10.3390/s25227033/s1, Figure S1: FDTD simulation sweep results of the excitation intensity confinement; Figure S2: Emission characteristics modification averaged across the Mie-void volume; Figure S3: Simulated far-field emission upward direction; Figure S4: Fluorescence image of Mie void immersed in the AF647 solution.; Supplementary Note S1: Calculations of Quasi-Normal Modes (QNMs) for Mie void; Figure S5: Normalized field intensity examples of physically meaningful (left) and unphysical artifact (right) QNMs.
