# Supplementary material for: Mie Voids for Single-Molecule Fluorescence Enhancement in Wavelength-Scale Detection Volumes
Source: Sensors (Basel). 2025 Nov 18;25(22):7033. doi: 10.3390/s25227033 (PMC12656445; doi:10.3390/s25227033)
Supplement: Supplementary file 1 [file sensors-25-07033-s001.zip › sensors-3908523-supplementary.pdf]

Supplementary Materials

Mie voids for single-molecule fluorescence enhancement in wavelength-scale detection volumes

Ivan Kuznetsov [1], Fedor Shuklin[1], Evgeny Ryabkov[1], Elena Barulina[1,2], Andrey Petukhov[1], Denis Baranov[1], Alexander Chernov[1,2], Aleksandr Barulin[1,*]

[1] Moscow Center for Advanced Studies, Kulakova Str. 20, 123592 Moscow, Russia
[2] Russian Quantum Center, Moscow, 121205, Russia
* Correspondence: alexbarulin73@gmail.com

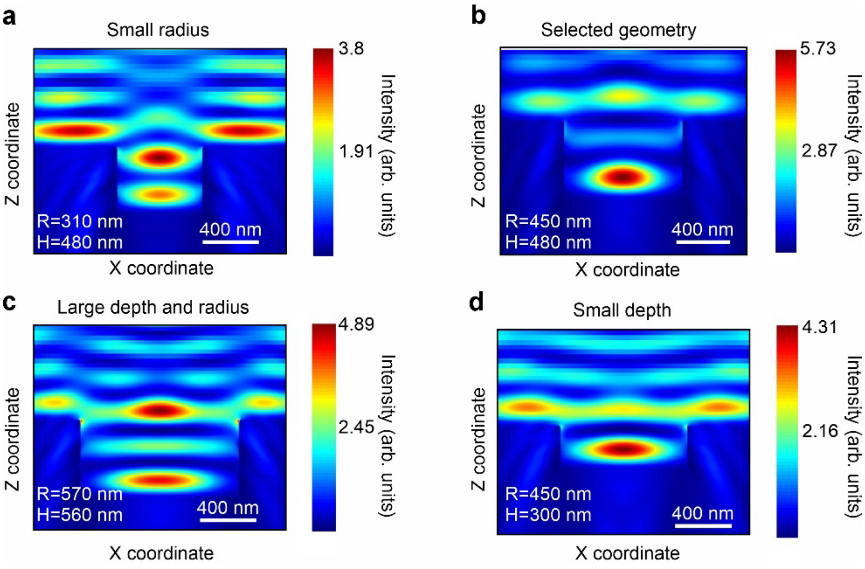

Figure S1. FDTD simulation sweep results of the excitation intensity confinement. Intensity distributions within the Mie voids in the *XZ* plane for the geometries of (a) radius (*R*) of 310 nm and depth (*H*) of 480 nm, (b) *R* of 450 nm and *H* of 480 nm, (c) *R* of 570 nm and *H* of 560 nm, and (d) *R* of 450 nm and *H* of 300 nm.

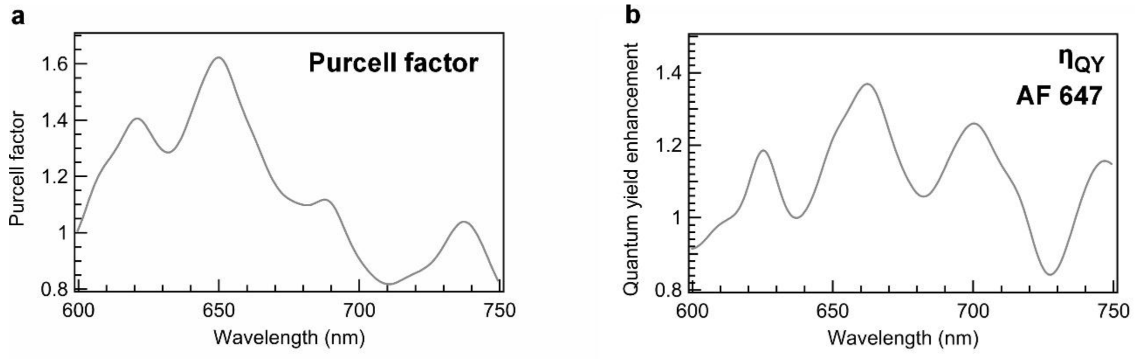

Figure S2. Emission characteristics modification averaged across the Mie-void volume. (a) Purcell factor dependence of the emitter as a function of wavelength. (b) Quantum yield enhancement of AF647.

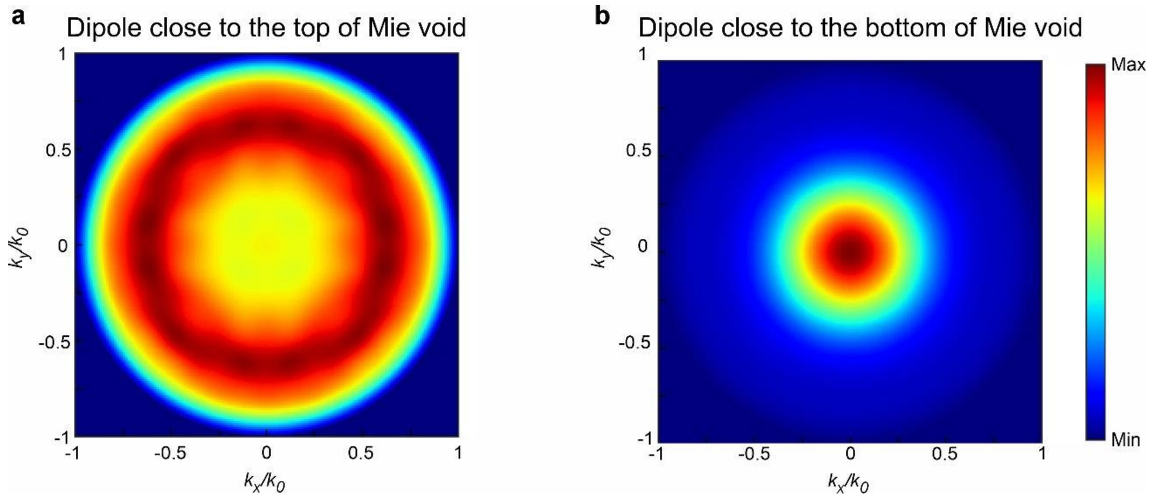

Figure S3. Simulated far-field emission upward direction. (a) Far-field emission pattern when an electric dipole is placed 20 nm below the entrance to the Mie void. (b) Far-field emission pattern when an electric dipole is placed 340 nm below the entrance to the Mie void (140 nm above the bottom of the Mie void).

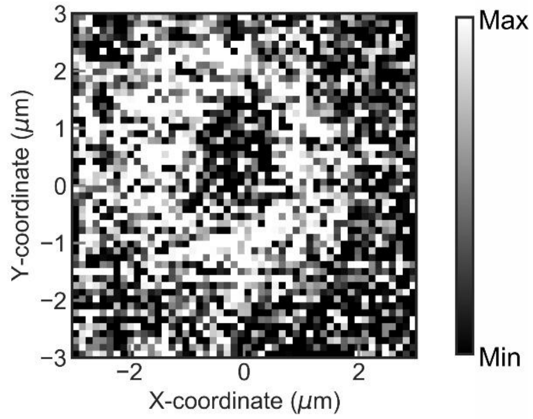

Figure S4. Fluorescence image of Mie void immersed in the AF647 solution. The dark points correspond to the signal of fluorophores at the surface of the bare silicon. Bright spots stem from the edges and the center of the Mie void.

Supplementary Note S1. Calculations of Quasi-Normal Modes (QNMs) for Mie void

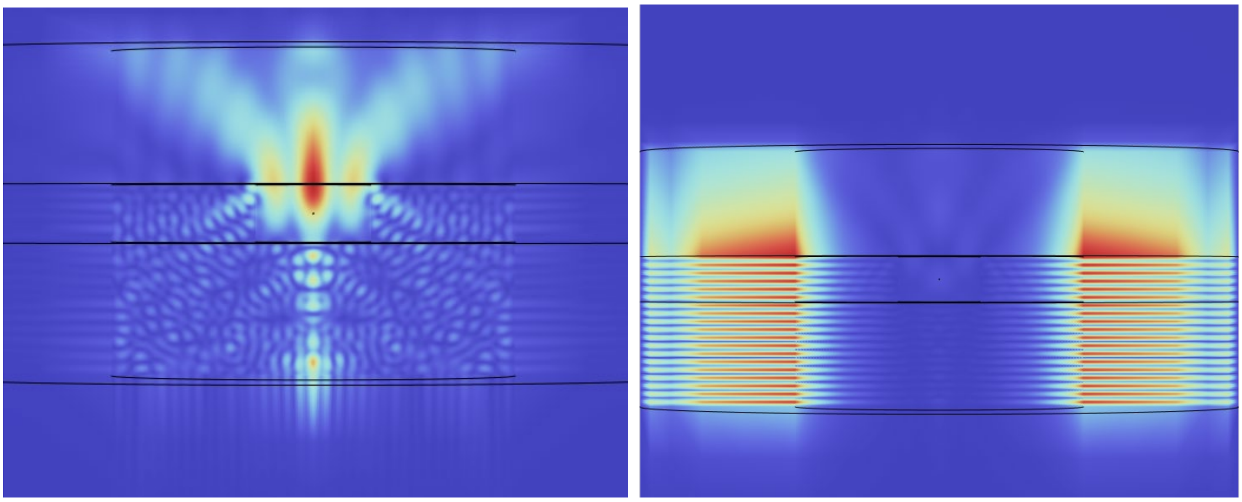

Figure S5. Normalized field intensity examples of physically meaningful (left) and unphysical artifact (right) QNMs. Physically meaningful mode exists at $^{\omega}/_{2\pi} = 451.7 + i8.1$ THz ($\lambda \approx$ 663 nm) with asimuthal mode number $m = 1$.

Here we outline the recipe for computing QNMs for a cylindrical Mie void (well in Silicon substrate). We use the COMSOL Multiphysics EWFD eigensolver to obtain eigenfrequencies and eigenmode fields. We configure a 2D axisymmetric model with a cylindrical domain bounded by PML. The size of the domain (well + free space region) and the thickness of the PML layer as well as PML scalings are set to $2\lambda_{max}$ and $3\lambda_{max}$ respectively, where λ_{max} = 700 nm; these values were defined such that a change in domain and/or PML resulted in a small (<1%) shift of the physically meaningful eigenfrequencies. Physically meaningful modes were separated from numerical artefacts by their localization. Physical modes must live inside the domain, and not inside PML or on the boundary between PML and the calculation domain, which is determined by comparison of energy ratio in the vicinity of PML and in the domain. PML scaling was selected such that it minimizes artifact reflections from inner and outer boundaries. We employ the QNM normalization procedure proposed in [S1], thus utilising PML once again.

Optical LDOS at a point r_0 can be calculated via the optical theorem as $\rho(r_0,\omega) = \frac{\omega}{\pi}\mathrm{Im}\,\{TrG(r_0, r_0, \omega)\}$, where $G(r_0, r_0, \omega)$ is Green tensor. In turn, the trace of the Green tensor can be calculated in two ways: by evaluating dipole emitter with dipole moment **d** at r_0 so $G(r_0, r_0, \omega) = 1/\omega\, E(r_0)\frac{d}{||d||^2}$, or with QNMs expansion [S1, S2]:

$$G(r_0, r_0, \omega) = G_{bg} + \sum_n \frac{E_n(r_0) \otimes E_n(r_0)}{\omega_n(\omega_n - \omega)} = G_{bg} + G_{QNM}\,.$$

In the latter case, G_{bg} is the background Green tensor, accounting for the background states near the interface without the void. The same Green's tensor gives the reference LDOS for the Purcell factor. This background Green's tensor can be obtained as the following integral [S3]:

$$G_{bg}(r_0, r_0, \omega) = \frac{\mathrm{i}}{8\pi}\int \frac{dk_{||}}{\sqrt{k_w^2 - k_{||}^2}}\left[r_s\,\hat{s} + r_p\,\frac{1}{k_w^2}\hat{p}\right],$$

where $\hat{s} = \frac{\hat{z} \times k_{||}}{|k_{||}|} \otimes \frac{\hat{z} \times k_{||}}{|k_{||}|}$ and $\hat{p} = \left(k_{||}\, \hat{z} \; + \; - k_{||} \sqrt{k_w^2 - k_{||}^2} \right) \otimes \left(k_{||}\, \hat{z} \; + \; - k_{||} \sqrt{k_w^2 - k_{||}^2} \right)$.

Thus, LDOS is

$$\rho(r_0, \omega) = \frac{\omega}{\pi} \left(\mathrm{Im}\{Tr\ G_{bg}(r_0, r_0, \omega)\} + \mathrm{Im}\{Tr\ G_{QNM}(r_0, r_0, \omega)\} \right) = \rho_{bg} + \rho_{QNM},$$

and Purcell factor is

$$F(r_0, \omega) = \frac{\rho(r_0, \omega)}{\rho_{bg}(r_0, \omega)} = 1 + \frac{\rho_{QNM}(r_0, \omega)}{\rho_{bg}(r_0, \omega)}.$$

For the practical calculations, we do not need to sum over QNMs with real parts of their frequencies lying within the detection window (~420-490 THz). Calculation with QNMs allows for relatively simple averaging of the Purcell factor over the void. Similarly, one can calculate Purcell enhancement with the former Green's tensor definition, by evaluation dipole emission power P with and without the void (dipole at r_0), thus:

$$F(\omega) = P(\omega) \,/\, P_0\,(\omega).$$

This approach, while allowing us to bypass calculations of QNMs, makes it more difficult to average Purcell enhancement over the void, as it requires sampling many positions of the dipole source. Furthermore, calculation of QNMs allows for computing Q-factors of the modes and mode volumes, following the procedure in [S1].

Supplementary references:

[S1] Sauvan, Christophe et al. "Theory of the spontaneous optical emission of nanosize photonic and plasmon resonators." *Physical Review Letters* 110 23 (2013): 237401.

[S2] Philip Trøst Kristensen, Kathrin Herrmann, Francesco Intravaia, and Kurt Busch, "Modeling electromagnetic resonators using quasinormal modes," Adv. Opt. Photon. 12, 612-708 (2020)

[S3] Novotny L, Hecht B. *Principles of Nano-Optics*. 2nd ed. Cambridge University Press; 2012., chapter 10
